# The Quality of Life in Patients With Implantable Cardiac Devices: A Single-Center Cross-Sectional Study

**DOI:** 10.7759/cureus.18542

**Published:** 2021-10-06

**Authors:** Wasna Alansari, Asmaa Mohammed, Rahaf Aljohani, Shahad Bakhashwain, Juan Jr S Manlangit, Faisal Al-Husayni, Nesreen Anajreah, Fahad Almehmadi, Amin Zagzoog, Atif AlQubbany

**Affiliations:** 1 College of Medicine, King Saud Bin Abdulaziz University for Health Sciences, Jeddah, SAU; 2 Internal Medicine, King Abdullah International Medical Research Center, Jeddah, SAU; 3 Internal Medicine, National Guard Hospital, King Abdulaziz Medical City, Jeddah, SAU; 4 Nursing, King Faisal Cardiac Center, National Guard Hospital, King Abdulaziz Medical City, Jeddah, SAU; 5 Medicine, King Saud Bin Abdulaziz University for Health Sciences, Jeddah, SAU; 6 Cardiology, King Faisal Cardiac Center, National Guard Hospital, King Abdulaziz Medical City, Jeddah, SAU

**Keywords:** inventory of depression and anxiety symptoms, idas, psychological impact, adjustment disorder, implantable cardiac device, quality of life

## Abstract

Background

Cardiovascular diseases (CVDs) and their complications are one of the most common causes of death worldwide. Implantable cardiac assistive devices (CADs) play a significant role in preventing dreadful outcomes, and the complication rate of these implanting procedures is minimal. These cardiac devices require some adaptation and could affect the patients’ quality of life psychosocially and financially. This study is aimed to identify the impact of implantable cardiac assistive devices on patients’ quality of life in the National Guard Hospital, Jeddah, Saudi Arabia.

Methods

This is an observational cross-sectional questionnaire-based study. It was conducted on patients who underwent cardiac assistive device implantation in National Guard Hospital. The patients were interviewed face-to-face and were requested to fill the Implanted Device Adjustment Scale (IDAS). Descriptive statistics were carried out. Chi-square test for independence was conducted to examine the associations between qualitative variables with the level of significance was taken as p-value <0.05.

Results

There was a statistically significant association between IDAS score and gender (p=0.03), monthly income (p=0.009), and type of cardiac implantation device (p=0.041). Females with an implantable cardiac defibrillator (ICD) and individuals with low socioeconomic status reported alongside divorced participants have higher IDAS scores, which correlates to worse adjustment. However, most of our patients scored 21-50 in IDAS score, which indicates a mild psychosocial effect after the cardiac assistive device implantation.

Conclusion

This study confirmed that most individuals adjust positively to implanted devices. It showed a significant association of gender, type of device, monthly income, and duration. Attention must be drawn to females and divorced patients in regards to psychological and emotional support.

## Introduction

Cardiovascular diseases (CVDs) are a broad term for conditions that are mainly affecting the heart and the blood vessels. They are serious illnesses that affect people of a variety of ages and races. According to the World Health Organization's latest statistics, 31% of all global mortality were due to CVDs, accounting for almost 17.7 million death making CVDs the leading cause of mortality [1]. Some factors increase the risk of CVDs, such as obesity, hypercholesterolemia, and diabetes mellitus [2]. The effect of these diseases and management may vary between different age groups and conditions.

Despite the advances in the medical therapy of CVDs, some conditions require and intervention with cardiac assistive devices (CADs) to decrease the mortality and morbidity rates [3]. Most of the patients that require implantation suffer from arrhythmias or conduction abnormalities [3]. Rapid intervention by implanting CADs may prevent sudden cardiac arrests.

There are three different types of implantable CADs. First of all, the permanent pacemaker (PPM) regulates heart rhythm by generating electrical pulses to keep the heart beating correctly, and it is indicated for patients who suffer from a second- or third-degree atrioventricular block and alternating bundle branch block [4]. Furthermore, an implantable cardiac defibrillator (ICD) keeps track of the patient's heart rate and delivers electrical shocks automatically and internally to reset the cardiac rhythm. It is commonly indicated for patients prone to sudden death due to sustained ventricular fibrillation, low ejection fraction, and fatal arrhythmias [4]. Finally, Cardiac Resynchronization Therapy (CRT) is a modality of cardiac pacing used in patients with heart failure, which consists of two modalities: CRT-pacemaker (CRT-P) and CRT-implantable cardioverter-defibrillator (CRT-D) [4]. While the implantation of the cardiac devices improves the patients' lives, these procedures carry the risk of complications affecting the life quality.

Quality of life (QoL) is a broad term that can be affected by multiple aspects other than a patient's general health status. These aspects involve the physical, psychosocial, and financial state of the patient. The assessment of QoL in patients with implantable CADs has been increasingly recognized as an essential aspect of patients' well-being. A study by Kaya et al. assessed the cardiovascular patient's experience of living with a pacemaker, which showed that such patients complained of limitations in their social life and other activities after the implantation [5]. Moreover, patients with CADs have a higher level of anxiety and depression [6]. However, there is a paucity of literature discussing the financial challenges in patients with CADs.

Herein, this study aims to identify the psychological impact of CADs implantations on patients' QoF in Saudi Arabia. The study intends to understand the possible influencers such as gender, financial, and marital status on patients' well-being with implantable CADs.

## Materials and methods

Study design and participants

This is a cross-sectional study conducted in the National Guard Hospital in Jeddah, Saudi Arabia. The data collection started on 1 February 2020 for a duration of 4 weeks. The study included all patients who were over the age of 18 years old. Patients who had their CAD implantation in a hospital other than National Guard Hospital were excluded. 

Sample size and sampling technique

The study utilized Roasoft Software to calculate the sample size. We set a 5% margin of error and a confidence level of 95%. The number of patients who had CADs implantation in the National guard was 266 individuals with an estimated response rate of 50%. The calculated sample size was 143 participants. The method sampling was a non-probability convenient sampling technique.

Materials and data collection process

An interview to answer a questionnaire was done to all included patients in National Guard Hospital, Jeddah, Saudi Arabia. It. The data were collected by interviewing the patients personally. The questionnaire consists of demographic characteristics and questions focusing on psychosocial and finical aspects. The psychological aspects were measured using the implanted Device Adjustment Scale (IDAS), which is a 21-item, 5-point Likert-type scale (strongly agree to disagree strongly) [7]. Scores can range from 21 to 105. If the responder scored between 81-105, he\she would consider being severely affected by the implantation. In contrast, if the responder scored between 21-50, he\she will be considered mildly affected, and if the responder scored between 51-80, he\she would be considered moderately affected. 

Data analysis

Data will be entered and analyzed using IBM SPSS version 21 (IBM Corp., Released 2012. IBM SPSS Statistics for Windows, Version 21.0. Armonk, NY: IBM Corp.) Demographic data will be summarized by frequency and percentage, mean, standard deviation. Chi-square and the t-test were used to determine differences in psychosocial (IDAS Score) and financial burden between groups in demographic variables. The Pearson correlation coefficient will be calculated for the dependent variable (IDAS score). Chi-square test for independence was conducted to examine the associations between qualitative variables with the level of significance was taken as P-value <0.05.

 

Ethical Approval:

The study received an ethical approval from King Abdullah International Medical Research Center (SP18/294/J).

## Results

A total of 111 patients completed the questionnaire. Table 1 shows the demographic characteristics of the participants. The mean age was 62.3+13.8 years, with males representing the majority (73.9%). A monthly income of less than 800 United States Dollars (USD) was observed in 51 (45.9%) participants, while 28 (25.2%) participants had a monthly income of more than 2500 USD.

**Table 1:** Demographic characteristics of patients with implantable cardiac assistive devices in Saudi Arabia (N = 111). USD: United States Dollar.

Demographic characteristics	Frequency (n = 111)	Percentage
Age (in years)		
18-30	4	3.6
41-50	18	16.2
51-60	30	27.0
61-70	27	24.3
Above 70	32	28.8
Gender		
Male	82	73.9
Female	29	26.1
Marital status		
Single	7	6.3
Married	84	75.7
Widowed	15	13.5
Divorced	5	4.5
Occupational status		
Retired	70	63.1
Unemployed	32	28.8
Employed or still working	6	5.4
Student	3	2.7
Monthly income		
Less than 800 USD	51	45.9
800-2500 USD	32	28.8
More than 2500 USD	28	25.2

The most common implantable device was an ICD in 50 (45%) patients, while CRT was used in only 7 (6.3%) patients. Moreover, 30 (27%) patients had PPM, and 24 (21.6%) patients had CRT-D.

The duration of CAD implantation is demonstrated in Table 2. Only two (1.8%) patients had the device for more than 36 months. The majority (29.7%) had the device for a duration between 13 and 24 months.

**Table 2:** Implantation duration for patients cardiac assistive devices (N=111).

Duration of cardiac device implantation	Frequency	Percentage
5-12 months	14	12.6
13-24 months	33	29.7
25-36 months	15	13.5
37 months and above	2	1.8
Unknown	47	42.3

Regarding IDAS scores, the total adjustment mean score was 36.78+9.54 (range 21-105). The scoring system showed that 101 (91.0 %) patients were having mild adjustment (21-50), while 10 (9%) patients had moderate adjustment scores, and none of the patients represented a severe adjustment score of 81-105.

Table 3 reveals the correlation between the IDAS score and participants’ demographic characteristics. The result of the t-test indicates that male patients with heart implantation device had significantly lower IDAS score (M=35.39, SD 9.08) compared to female patients (M=40.72, SD 9.87), t=-2.25, p=0.014). In chi-square test result, widowed and married were significantly more likely to have lower IDAS score compared to divorce (X2 = 3.2, p=0.05), and those with a monthly income of more than 2500 USD were significantly more likely to have lower IDAS compared to those with lower than 2500 USD monthly income (X2 = 4.4, p=0.05).

**Table 3:** Table 3: Differences in IDAS score when grouped according to demographic characteristics (N=111). USD: United States Dollar; IDAS: Implanted Device Adjustment Scale. *The mean difference is significant at the 0.05 level (t-test). **The mean difference is significant at the 0.05 level (chi-square test).

Gender	n	Mean	SD	t-test	p-value
Male	82	35.3902*	9.08	-2.55	0.014
Female	29	40.7241*	9.87		
Civil status					
Single	7	40.1429	7.26702	3.268	0.024
Married	84	36.6905**	10.04565		
Widowed	15	32.4667**	4.68838		
Divorced	5	46.6000**	6.69328		
Monthly income					
Less than 800 USD	51	39.5490	10.57793	4.434	.014
800-2500 USD	32	35.1875	6.34715		
More than 2500 USD	28	33.5714	9.46114		

Table 4 demonstrates the Pearson correlation coefficient of IDAS score as a dependent variable with other variables. There was a statistically significant association between IDAS score to gender (p=0.03), monthly income (p=0.009), and type of heart implantation device (p=0.041). The gender (beta=0.352) accounted for most of the variability in the IDAS score, followed by monthly income (beta=-0.328) and type of implantation device (beta=-0.253). The overall regression equation was highly significant, p<.01, and the 28% of the IDAS score variance was explained by the combined effect of gender, monthly income, and type of heart implantation device. The remaining 72% of unexplained variation to IDAS score will require further study.

**Table 4:** Table 4: Pearson correlation coefficient of IDAS score as a dependent variable and other variables among patients with implantable cardiac assistive devices. IDAS: Implanted Device Adjustment Scale.

	Dependent variable (IDAS Score)		
Independent variables	Unstandardized coefficients B	Standardized coefficients B	p-value
Gender	6.706	0.353	0.033
Age	-0.024	-0.042	0.777
Civil status	-0.092	-0.007	0.966
Occupation	-0.273	-0.063	0.689
Monthly income	-3.380	-0.328	0.009
Type of heart implantation device	-1.837	-0.253	0.041
Duration of implants (months)	0.002	0.084	0.473
Overall ANOVA: R^2^=0.282, F=176.117, p=0.006			

## Discussion

The study aimed to assess the effect of implantable CADs on patients' QoL. Patients who are females, not married, or have a low income are at higher risk of being affected psychologically by the device implantation. The device type and the duration of having it did not show a significant effect on the adjustment scale. However, the combination of gender, monthly income, and type of heart implantation device showed a significantly higher IDAS score. In this study, we selected the IDAS questionnaire due to its simplicity and applicability. Although it is a very uncomplicated questionnaire, its design does not negatively affect its reliability and usefulness.

In our study, the participants' mean age is comparable to other studies with a similar topic [8,9]. Also, our data showed that the majority of the participants were males, which goes in concordance to other studies that demonstrated that males have a higher probability of getting atrioventricular block (AV), structure heart disease, and ischemia cardiomyopathy that needed ICD implantation, compared to female patients [10,11]. Moreover, our results revealed a significant increase in the need for cardiac device implantation in people who are 70 years old and above compared to the other age groups, with a percentage of 28.8%. A similar finding was observed as people who are 65 years old and above have higher ICD implantation compared to other age groups [12,13]. On the other hand, patients' co-morbidities play a significant role in cardiac device implantation. For instance, patients with moderate to severe renal impairment and a history of life-threatening ventricular arrhythmias did not show benefits from ICD implantation [14,15].

Patients' QoL is a crucial variable to assess the value of a particular treatment. Nowadays, treatment goals have expanded not only to extend a patient's life but to alleviate disabling conditions, and patients' suffer. Multiple studies included QoL as an outcome of treatment [16,17]. Our findings showed encouraging results, as only 9% of the patients had moderate adjustment scores, and none of the patients had severe adjustment scores. Nonetheless, females with ICD had higher IDAS scores, which can be explained by their concern about appearance as reported by Marshall et al. [18]. 

Interestingly, married and widowed participants have significantly lower IDAS scores when compared to their divorced peers. Depression has been reported to be lower in patients who were married in terms of heart failure, while widowed patients were more likely to have a worse cardiovascular risk profile and one-year outcome [19,20]. Flemme et al. revealed that patients with ICDs tend to use coping strategies early after the implantation without significant efficacy [21]. Moreover, The authors hypothesized that nurses' role in supporting and counseling patients about stress and anxiety management might help them accept the situation.

Finally, we found a strong association between low socioeconomic status and cardiac assisted device implantation as the majority of the participants were within the low socioeconomic status group with an average monthly income of 800 USD. A Danish nationwide study reviewing the same association between socioeconomic status and the cardiac assisted devices resulted in an opposite finding. It stated that individuals with low socioeconomic status and public insurance were associated with lower device implantation [22]. Concerning the difference between our results and the other studies, it could be related to government health coverage in Saudi Arabia.

Limitations

The study is limited to a small sample size and a single hospital. It was also limited by the convenience sampling method, which introduced a possible selection bias.

## Conclusions

Most of the patients had good QoL after implanting a CAD as none of the patients have severe adjustment, and only 9% had a moderate adjustment. Females and divorced were at higher risk of having a severe adjustment. Also, there is a strong association between participants and low socioeconomic status. Intensifying the support to the patients who have a higher risk of severe adjustment after CADs implantation is highly recommended.
